# Natural infection by the protozoan *Leptomonas wallacei* impacts the morphology, physiology, reproduction, and lifespan of the insect *Oncopeltus fasciatus*

**DOI:** 10.1038/s41598-019-53678-1

**Published:** 2019-11-25

**Authors:** Luiz Ricardo C. Vasconcellos, Luiz Max F. Carvalho, Fernanda A. M. Silveira, Inês C. Gonçalves, Felipe S. Coelho, Octávio A. C. Talyuli, Thiago L. Alves e Silva, Leonardo S. Bastos, Marcos H. F. Sorgine, Leonan A. Reis, Felipe A. Dias, Claudio J. Struchiner, Felipe Gazos-Lopes, Angela H. Lopes

**Affiliations:** 1 Instituto de Microbiologia Paulo de Góes, Universidade Federal do Rio de Janeiro, Rio de Janeiro, RJ 21941-590 Brazil; 2 Escola Nacional de Saúde Pública (ENSP). Programa de Computação Científica (PROCC), Fundação Oswaldo Cruz, Rio de Janeiro, RJ 21045-900 Brazil; 3 Institute of Evolutionary Biology, School of Biological Sciences, University of Edinburgh, Edinburgh, UK EH9 3JT; 4 Laboratory of Malaria and Vector Research, National Institute of Allergy and Infectious Diseases, National Institutes of Health, Rockville, Maryland 20852 USA; 5 Instituto de Bioquímica Médica Leopoldo de Meis, Universidade Federal do Rio de Janeiro, Rio de Janeiro, RJ 21941-590 Brazil; 6 Escola de Matemática Aplicada (EMAp), Fundação Getúlio Vargas, Rio de Janeiro, RJ 22250-900 Brazil

**Keywords:** Parasite host response, Entomology

## Abstract

Trypanosomatids are protozoan parasites that infect thousands of globally dispersed hosts, potentially affecting their physiology. Several species of trypanosomatids are commonly found in phytophagous insects. *Leptomonas wallacei* is a gut-restricted insect trypanosomatid only retrieved from *Oncopeltus fasciatus*. The insects get infected by coprophagy and transovum transmission of *L. wallacei* cysts. The main goal of the present study was to investigate the effects of a natural infection by *L. wallacei* on the hemipteran insect *O. fasciatus*, by comparing infected and uninfected individuals in a controlled environment. The *L. wallacei*-infected individuals showed reduced lifespan and morphological alterations. Also, we demonstrated a higher infection burden in females than in males. The infection caused by *L. wallacei* reduced host reproductive fitness by negatively impacting egg load, oviposition, and eclosion, and promoting an increase in egg reabsorption. Moreover, we associated the egg reabsorption observed in infected females, with a decrease in the intersex gene expression. Finally, we suggest alterations in population dynamics induced by *L. wallacei* infection using a mathematical model. Collectively, our findings demonstrated that *L. wallacei* infection negatively affected the physiology of *O. fasciatus*, which suggests that *L. wallacei* potentially has a vast ecological impact on host population growth.

## Introduction

Infections induce physiological alterations that can potentially impact host lifespan, development, reproduction, and behavior^1–4^. Combined, these phenotypical alterations may reduce the overall fitness and impair host development^5–7^. Such modifications usually arise in response to microorganism by-products or host adaptation to the infection, or both^8^. Thus, these host physiological alterations may directly or indirectly impact population development and shape their community structure in nature^9,10^. Despite several reports demonstrating disturbances in reproduction and mating behavior related to infection, little is known about the long-term negative impacts of such infections on the population dynamics in the ecosystem^11,12^.

Trypanosomatids are protozoans that parasitize all classes of vertebrates, several invertebrates (mostly insects), and plants^13–15^. *Leptomonas* is a genus of the family Typanosomatidae (order Trypanosomatida, class Kinetoplastea); this order is solely comprised of flagellated parasites^13,14^. A high percentage of trypanosomatids infect only insects, but the genera *Trypanosoma* and *Leishmania* are considered the most important ones because they cause severe illnesses in humans and often lead to the death of infected patients^15^. *Leptomonas wallacei* is a gut-restricted insect parasite that naturally infects the seed-eater *Oncopeltus fasciatus* (Hemiptera: Lygaeidae)^16–18^.

The hemipteran *O. fasciatus* has been used in seminal studies on embryology^19,20^, cytogenetics^21,22^, biochemistry^23,24^, nucleic acids^25^ and interaction with its natural or experimental trypanosomatid parasites^26–28^. Some of these studies date back to 1926^26,29^ when a natural infection of *O. fasciatus* by *Leptomonas* spp. was first described^26^. Later on, *O. fasciatus* was widely employed in molecular studies that intended to shed light on embryogenic and physiological aspects of this insect^30,31^, which has since been considered a pivotal model for studying evolutionary developmental biology^30^. RNA interference (RNAi) approaches have been successfully used to hamper expression and determine the influence of specific genes on sexual and morphological development on *O. fasciatus* species^32,33^. In addition, other studies have been conducted to better understand the relationship between *O. fasciatus* and trypanosomatids^16–18,34–38^.

Host responses to infections are often studied in artificial models rather than in natural conditions. Nevertheless, investigations of natural infections are preferable to elucidate their actual impact on host populations^39,40^. In general, infections caused by trypanosomatids affect insect physiology by reducing their reproductive capacity, impairing host development and locomotion, by modifying hosts behavior, and by increasing host mortality^41,42^. Since its first description, *L. wallacei* has not been considered pathogenic towards *O. fasciatus* in short-term infections. Conversely, no data is available for long-term infections^16^. The transmission of *L. wallacei* between insects occurs via the ingestion of cystic forms through coprophagy, as well as by transovum transference of cysts that adhere to the eggs during oviposition; i.e., the newly born nymphs are infected by probing the fecal droplets on eggshells^36^. It was common for the *O. fasciatus* individuals that we used in our laboratory to be naturally infected by *L. wallacei*^16^. Therefore, we generated another colony of *O. fasciatus*, which we raised from disinfected eggs. The novelty of the present study was to compare the *L. wallacei*-free and the naturally infected insects in a controlled environmental laboratory model to determine the long-term effects of natural infection. Hence, we demonstrated that a natural protozoan infection can significantly affect the insect host development and population growth, which could greatly impact the population dynamics of the host species.

## Results

### *Leptomonas wallacei* infection reduces *Oncopeltus fasciatus* lifespan

**Figure 1:**
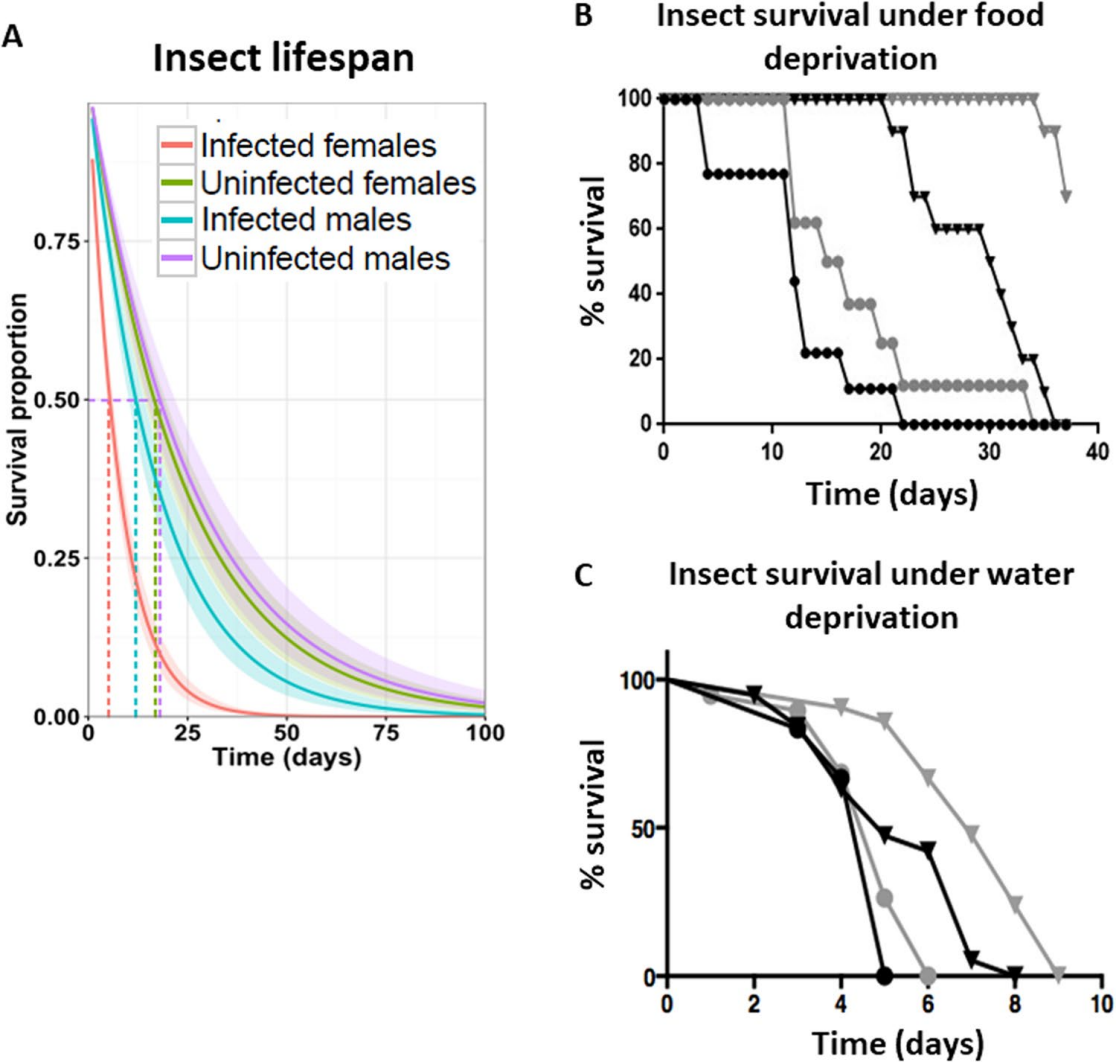
*Leptomonas wallacei* infection reduces *Oncopeltus fasciatus* survival. (**A**) Lifespan of adult insects, fed *ad libitum*, from infected and uninfected colony. Infected males, blue line (n = 12), uninfected males, purple line (n = 18), infected females, red line (n = 27) and uninfected females, green line (n = 18). Lifespan of adult insects maintained submitted to food (**B**) or water deprivation (C). Infected males, black circles (n = 10), uninfected males, black triangles (n = 10), infected females, gray circles (n = 10) and uninfected females, gray triangles (n = 10).

The study of natural infections may help to understand the burden of parasitic diseases on host fitness and population dynamics. Previously, we have demonstrated that *L. wallacei* is vertically transmitted and reaches 100% infection in *O. fasciatus* adults^38^. Here, we used that experimental model to evaluate the impact of *L. wallacei* natural infection on *O. fasciatus*, as compared to non-infected insects. First, we observed the effect of *L. wallacei* on *O. fasciatus* life expectancy. Notably, infected females were more affected by infection than males, as 50% of infected females showed a greatly reduced lifespan, i.e., they died twice as fast as the uninfected ones (Fig. 1A). Moreover, under stress conditions caused by food and water deprivation, infected insects died earlier in life than uninfected insects, although no difference in stress susceptibility between sexes was noted (Fig. 1B,C). These experiments demonstrated that infection impacts host survival in either normal situations or under stress conditions, with higher severity in females, but only in normal conditions. To evaluate the importance of infection status in insect development and mortality, we observed the insects from the time they hatched from eggs into first instar nymphs until they reached the adult stage. We observed a slight delay in time from fifth instar nymph to adult in infected insects, although there was no difference in life expectancy between infected and uninfected nymphs (Fig. S1). Therefore, *L. wallacei* infection also induces a delay in the development of *O. fasciatus*.

### *Leptomonas wallacei* induces morphometric alterations in *Oncopeltus fasciatus*

**Figure 2:**
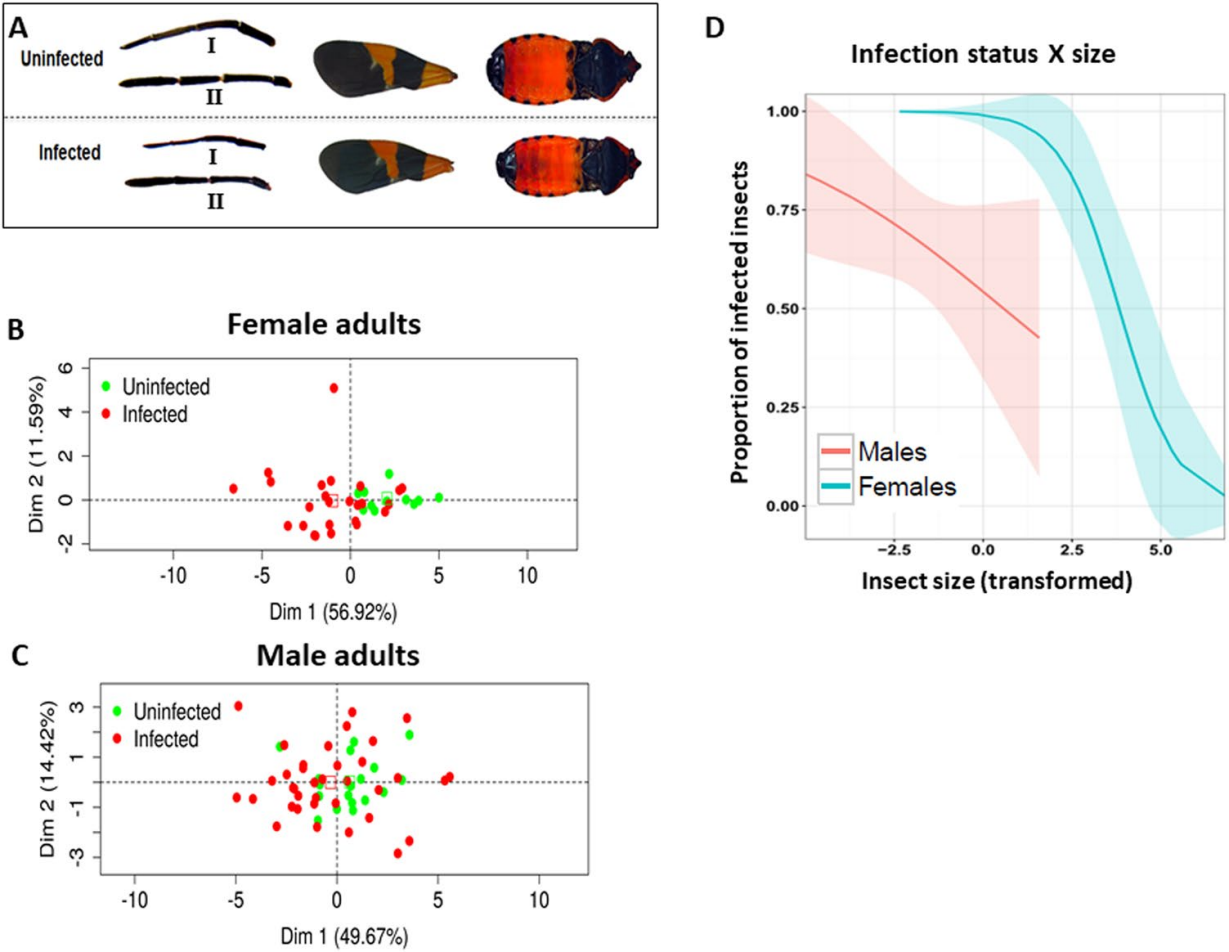
(**A**) *Leptomonas wallacei* infection induces host morphology alterations. Different body parts of uninfected (above) and infected (below) *Oncopeltus fasciatus* side by side for comparison (1- rostrum, 2- antenna, 3- forewing, and 4- body length). Principal components analysis (PCA) plots for the morphometric data in females (**B**) and males (**C**). All morphometric variables were transformed using the PCA, the first and second principal components were plotted, and each point graphically represents a sample (infected-red dots and uninfected-green dots). (**D**) Generalized linear model (GLM) prediction of the probability of being infected. Results of GLM prediction of the probability of being infected in response to the size principal component derived using the PCA for males and females in the adult dataset.

From the analysis of insect development, we observed severe morphologic alterations in infected insects that were rarely observed in uninfected insects (Fig. 2). To demonstrate these differences graphically, we devised a measurement of overall morphology to compare infected and uninfected insects (see Methods for details, Figs. S2 and S4, Table 1). We observed a significant reduction in the overall size of infected insects (Fig. 2A, see below). Despite the morphological alterations, no differences in weight were observed between infected and uninfected virgin adult insects, which demonstrated that the alterations in overall morphology did not result in weight loss (Fig. S2A). Since *L. wallacei* infection induces severe morphological alterations on *O. fasciatus*, we tried to distinguish the insects by their infection status by applying a principal component analysis (PCA) to morphological features. Scatter plots of the first two principal components (PCs), individually colored by infection status, are shown in Fig. 2B (females) and 2 C (males). For both groups (males and females), PCA resulted in the first PC that explained ~50% of the total variance and was positively correlated with all the original variables (more details in the supplementary text). These analyses showed that there is a clear separation between infected and uninfected females (Fig. 2D, Figs. S4A,B) in the morphometric (feature) space and that this separation is more evident, and thus able to distinguish females by their infection status (Fig. 2B). To clearly demonstrate the association between morphology and infection, we took the first PC as a combined measure to describe insect size and used this variable as a predictor of infection status in a binary generalized linear model (GLM). As shown in Fig. 2D, it was possible to detect a significant association between insect size and infection (see Fig. S4C for the same analysis in the nymph dataset). Moreover, it was possible to observe the association between insect size and infection status and more visibly demonstrate higher severity of alterations in infected females than in males, in the GLM analysis.

**Table 1:** Morphometric analysis of the insects.

*status*	Males			Females		
	Infected	Uninfected	*p*	Infected	Uninfected	*p*
weight (g)	0,04 (0,01)	0,04 (0,00)	0,0178	0,06 (0,01)	0,06 (0,01)	0,0993
lenght (mm)	10,60 (0,51)	10,77 (0,43)	0,165	11,66 (0,56)	12,20 (0,31)	< 0,001
hindwing lenght (mm)	7,85 (0,43)	8,01 (0,39)	0,1303	8,54 (0,43)	9,00 (0,28	< 0,001
forewing lenght (mm)	9,69 (0,53)	9,85 (0,48)	0,2243	10,70 (0,57)	11,21 (0,40)	0,002
hindwing area (mm^2^)	17,37 (2,04)	18,71 (1,54)	0,0051	19,98 (2,37)	23,58 (1,56)	< 0,001
forewing area (mm^2^)	19,86 (2,06)	20,82 (1,72)	0,0533	23,96 (2,43)	27,09 (2,07)	< 0,001
rostrum lenght (mm)	5,26 (0,43)	5,33 (0,39)	0,5374	6,03 (0,56)	6,10 (0,31)	0,5964
antenna lenght (mm)	6,29 (0,30)	6,53 (0,33)	0,0051	6,55 (0,34)	7,24 (0,28)	< 0,001
abdomen width (mm)	3,69 (0,23)	3,78 (0,22)	0,1221	4,13 (0,25)	4,33 (0,17)	0,0043
leg lenght (mm)	7,11 (0,83)	7,65 (0,70)	0,0079	7,50 (0,96)	7,84 (0,62)	0,181
insect area (mm^2^)	30,35 (2,96)	31,92 (2,43)	0,026	38,15 (3,51)	41,23 (2,20)	0,0013

Virgin insects (one week after becoming adults) were collected from both colonies, separated by sex and measured. The expressed values are the average values obtained for each parameter. In parenthesis is the standard deviation value for each data group. The weight, body size, total area of the body, membranous wing and hemi-elytra size, membranous wing and hemi-elytra area, rostrum and median leg size, abdomen width and antenna size of the insects were measured. The weight of the insects was obtained using a precision scale and the measurements were obtained using the software Analyzing Digital Images (Museum of Science, Boston). The morphometric variables between the groups were performed using unpaired *t* test.

### Female insects are more susceptible to *Leptomonas wallacei* infection than males

**Figure 3:**
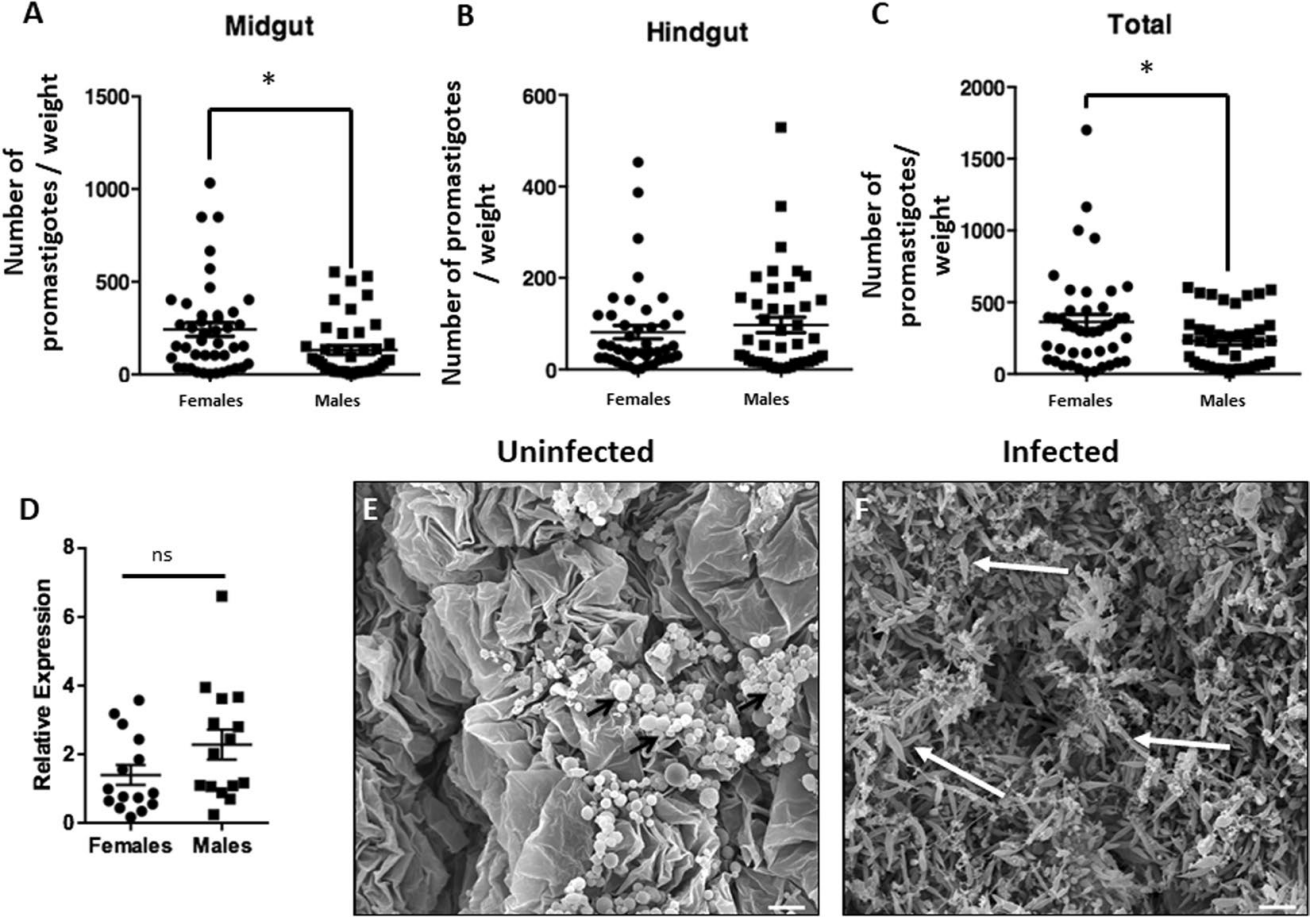
Parasite number in the gut of infected *Oncopeltus fasciatus*. Insect intestine was separated into the midgut and hindgut and promastigote forms counted in a Neubauer chamber (**A,B**). The overall number of promastigotes was obtained using the sum of the number of parasites in the midgut and hindgut (**C**). Insects were collected from the infected colony and their alimentary tracts were dissected for qPCR analysis for comparative *L. wallacei* gene expression (**D**). The hindgut was dissected from an uninfected or infected insect and observed by scanning electron microscopy (**E,F**). No flagellates were observed in the gut of an uninfected insect (**E**), whereas the gut of an infected insect shows a massive presence of flagellates (**F**). Black short arrows show uric acid spherules (**E**)^66^ and white long arrows highlight *L. wallacei* promastigotes (**E**). Scale bars = 10 µm.

To investigate sex differences in lifespan and the impact of infection on morphological features, we evaluated the presence of parasites in the intestine of males and females. Through the analysis of the relative expression of a *L. wallacei*-specific sequence (16 S rRNA gene), no difference was observed between males and females (Fig. 3D). Nevertheless, promastigote counts were higher in females than in males and the difference observed was noticeably located in the midgut, i.e., the main site of *L. wallacei* infection, and no difference in the hindgut number of parasites was observed (Figs. 3A–C and S5). To visualize the infection micro-environment, we prepared scanning electron micrographs of insect midguts and observed massive amounts of parasites attached to the whole gut wall in infected insects, whereas no parasites and intact intestinal structures were observed in uninfected ones (Fig. 3E,F). Therefore, our images clearly demonstrated that *L. wallacei* induces gut micro-environment alteration when established in the infection site. Moreover, there was no difference in *L. wallacei* relative gene expression between males and females. Despite that, females present more *L. wallacei* promastigotes in the intestine reflected by a robust difference in promastigote forms in the midgut (Fig. 3C).

### *Leptomonas wallacei* infection reduces *Oncopeltus fasciatus* reproductive fitness, downregulating *intersex* gene expression

**Figure 4:**
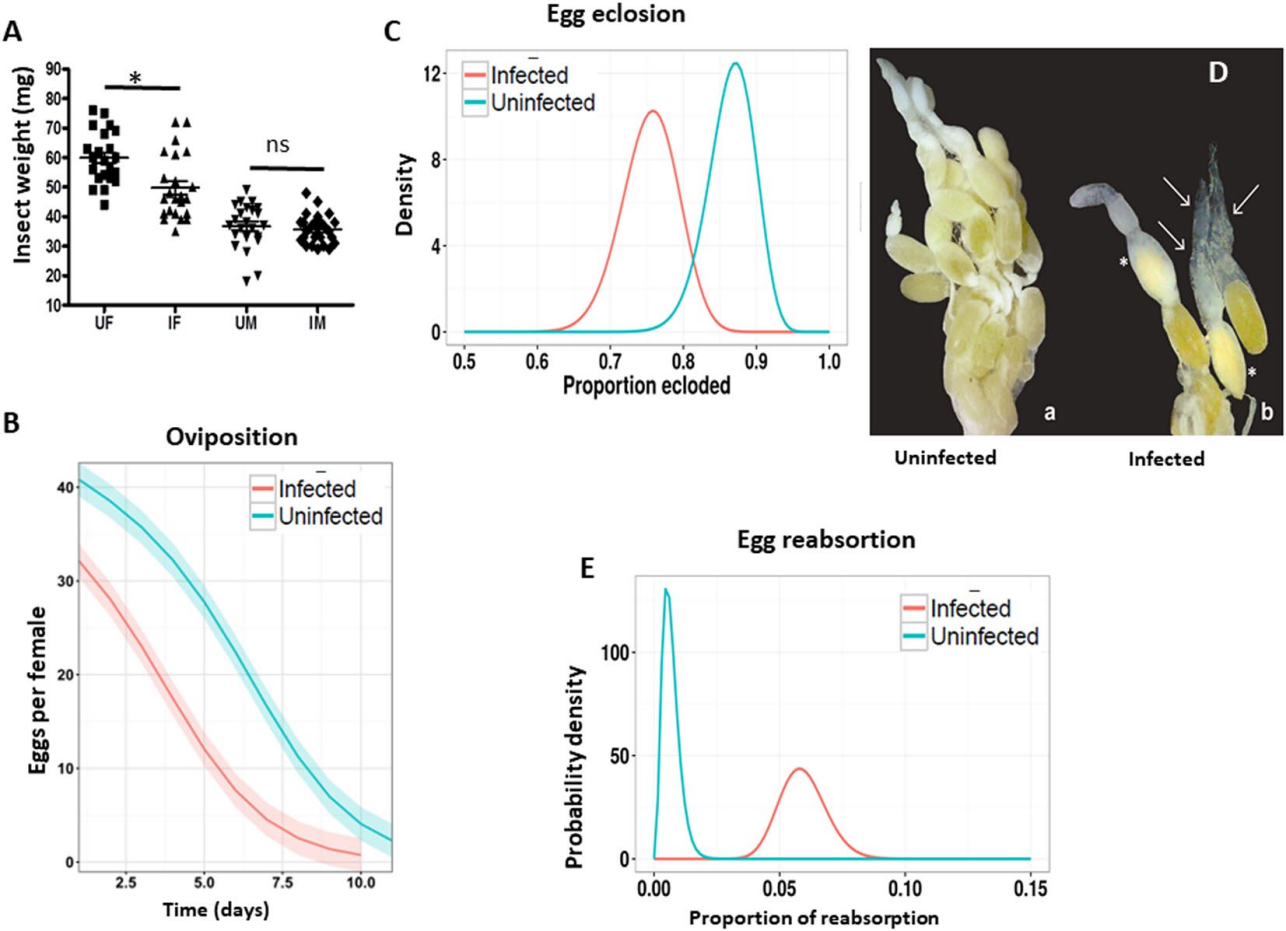
*Leptomonas wallacei* infection induces reproductive fitness reduction in *Oncopeltus fasciatus*. (**A**) Reproductively active adult females and males were collected and weighed using a precision balance (UF-uninfected female, IF-infected female, UM-uninfected male, IM- infected male) * represent significant differences in the one-way ANOVA test. (**B**) Oviposition from both colonies was monitored daily for two weeks and the average daily ovipostion was predicted using the zero-inflated Poisson model. Generalized linear model formulation was used with the R statistical pscl package to compute environmental variables. (Uninfected n = 17 and infected n = 20). (**C**) Egg eclosion was determined by counting the number of ecloded eggs laid from both colonies. The x-axis shows the estimated proportion of eclosion and the y-axis shows the probable density (uninfected n = 90 and infected n = 120). (**D**) Representative images of the morphology of female ovaries from an uninfected or infected female. (**E**) Egg reabsorption was observed by classifying and counting the number of eggs in dissected females. The x-axis shows the estimated proportion of reabsorption and the y-axis shows the probability density (uninfected n = 30 and infected n = 30).

In the present study, we observed that females were more susceptible than males in terms of changes to their morphology and lifespan. This led us to wonder if this higher susceptibility was related to the cost of carrying eggs during reproduction. We thus tested if *L. wallacei* infection also impacted *O. fasciatus* reproduction. Reproductively active infected females were lighter than the uninfected ones, whereas no difference in weight was observed between males (Fig. 4A). Because females need to carry eggs, we investigated if the observed weight loss was associated with a decrease in reproductive output (Figs. 4B,C, S6A,B). Uninfected females laid more eggs than infected ones and the decrease in the number of eggs laid overtime was slower in uninfected than in infected females (Figs. 4B and S6A for absolute values). We observed a ~27% reduction in laid eggs from infected insects in comparison to uninfected ones. Furthermore, the eclosion rate of eggs laid from uninfected insects was higher than from infected insects (Fig. 4C and Table S1 in data S1, for overall values).

To investigate the reason for the reduction in the number of eggs laid and the eclosion of eggs in infected insects, we evaluated the eggs in the abdomen of females from both colonies. The average egg load of uninfected females is 30 eggs; infected females had an average of 23 eggs. Infection by *L. wallacei* reduced the egg load of *O. fasciatus* by almost 25% (S2 Table in S1 data). To verify if there were differences between these loaded eggs, we also evaluated the morphology of eggs from uninfected and infected insects (Figs. 4D and S6B). We observed that ~2.5% of the eggs from infected females showed characteristics of reabsorption, whereas less than 0.5% of the eggs from uninfected females showed these characteristics (Fig. 4E).

**Figure 5:**
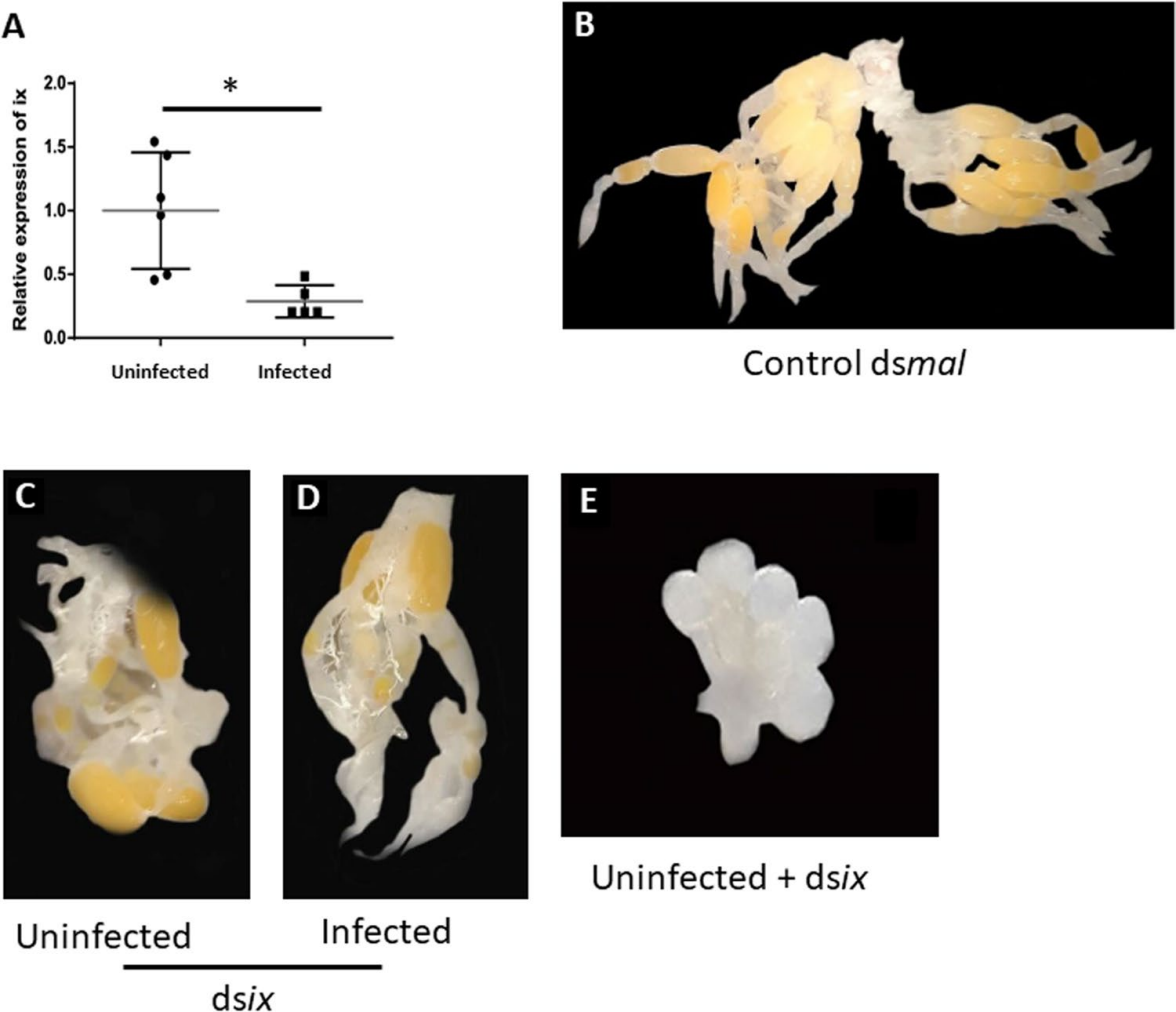
*L. wallacei* infection impacts on *O. fasciatus* intersex (ix) gene expression reproductive organs resulting in reproductive fitness loss. (**A**) Ovarian *ix* gene expression was assessed by qPCR in uninfected and infected females. (**B**) Representative image of ovary of an uninfected insect subjected to control ds*mal* protocol; showing normal ovary morphology. Reproductively active uninfected (**C**) and infected (**D**) insects silenced with ds*ix*. (**E**) Representative image of an ovary from an uninfected female subjected to RNAi with ds*ix*; showing an atrophied ovary morphology. Representative of two independent experiments.

After demonstrating the reproductive impact of *L. wallacei* infection on *O. fasciatus* development and reproduction fitness, we investigated the influence of the parasite and infection on the expression of a gene related to all these parameters, the intersex gene (*ix*). When *Ix* expression was evaluated between both colonies, uninfected individuals expressed higher levels of *Ix* than the infected ones (Fig. 5A). In addition, we applied RNAi for the *Ix* gene and the females subjected to *Ix* RNAi protocol showed the same egg reabsorption rates as observed in infected females, albeit the control RNAi had no effect on egg formation (Fig. 5B–D). After demonstrating that the *Ix* gene was essential for ovarian maturation, insects that were subjected to ds*ix* also showed ovarian atrophy (Fig. 5E).

### *Leptomonas wallacei* infection impacts *Oncopeltus fasciatus* population dynamics

**Figure 6:**
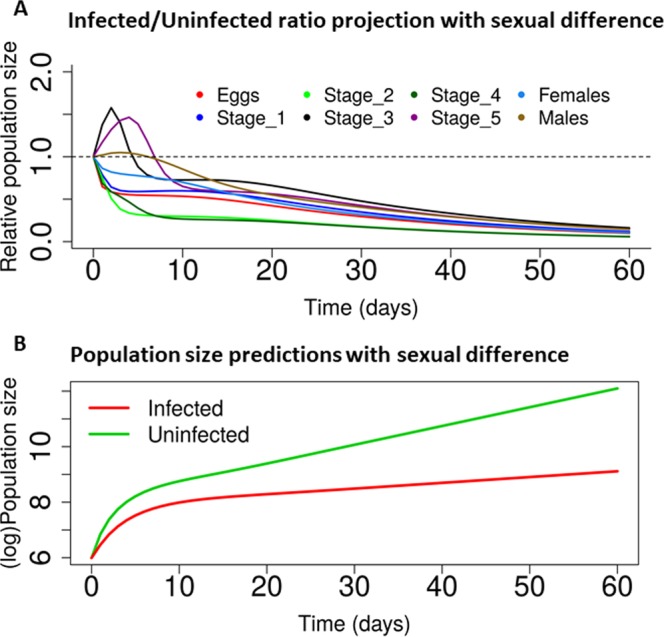
*L. wallacei* affects *Oncopeltus fasciatus* population dynamics with sex-bias. (**A**) Development, lifespan, and reproductive data from infected and uninfected colonies of *O. fasciatus* were combined to model the relative population size of each life stage in the population dynamics considering the sexual differences. (**B**) Population projections for uninfected or infected colonies. Even if infection impacts the population, it is still shown to grow exponentially.

To assess the population-level impact of natural infection by *L. wallacei* on its host, we proposed a simple ordinary differential equation-based mathematical model to obtain short-term population projections for infected and uninfected colonies. We used the data and estimates observed in this study to create two separate sets of parameters, one for each scenario (infected and uninfected) and using an initial population size N(0) = 400. First, we obtained the ratio of each stage trajectory in the infected scenario relative to its counterpart in the reference scenario. This offered unit-free trajectories, which we can then be studied to determine the impact of infection on the growth of each life stage. Figure 6A shows the results of this analysis and it is notable that the ratios tend to reach zero as time progresses. Interestingly, there was also a temporary increase in the number of females in the infected population, likely driven by the transient rise in nymphs of the fourth instar. This result is most likely a consequence of sampling error in the measurements of development rates (which indicated higher rates of transitions from the third instar to the fourth instar in the infected group), although the possibility that the molting rates may be higher in infected insects still requires investigation. The long-term behavior of the model, however, indicated a clear reduction in population size in the infected scenario, which was confirmed by the overall population projections in Fig. 6B. This analysis showed exponential growth as expected since, apart from infection, all conditions for insect development and reproduction were optimal. We draw the reader’s attention, to the striking difference in population projections between infected and uninfected groups, a gap that increases as time progresses. The results shown in Fig. 6A,B account for the differential mortality between males and females, as this is an important finding of the present study. Similar projections using a combined mortality rate for adults, i.e., a model without sexual differentiation, are presented in Fig. S7 and the gap between the infected and uninfected population albeit present, seemed to be smaller than the one from the model that accounts for differential mortality.

## Discussion

The current study presents the broad range of long-term effects of a natural *L. wallacei* infection on *O. fasciatus* fitness, including alterations in life expectancy and gene expression that culminate in reproductive deficits. Here, we propose an association between the morphology and infection status on the effects of this natural on host trypanosomatid infection physiology, as demonstrated by several parameters. In addition, we demonstrated an infection sexual bias, being females more susceptible than males. On average, infected females survived for half the period of time than uninfected females. Several reports have demonstrated that infections caused by insect pathogens impact the insect’s lifespan, although sex-biases were not described^45–48^.

Infected adults survived for a shorter period of time than the uninfected ones, even though the same was not observed in the early stage nymphs. This might be explained by the increased rate of *L. wallacei* infection over time, as previously demonstrated by our group. Here, we also observed the reduced lifespan of insects subjected to stress conditions and the higher susceptibility of infected insects in terms of a lower life expectancy than uninfected, as has also been demonstrated in several trypanosomatid-host interaction models that have considered sub-pathogens^49^. In nature, insects sometimes live under stress conditions and our results suggest that infected insects, males and females with no sex-bias, were more susceptible to water and food deprivation than uninfected individuals, which might impair the ability of infected hosts to persist in the environment.

In the present study, a massive amount of *L. wallacei* was attached to the gut wall of the host, which might cause disturbances of the digestive physiology and interfere with the normal functioning of this organ and with nourishment. This may explain why infected insects had shorter life expectancies in our model. Similar scenario has been reported for vector interactions with other trypanosomatids, such as *Blastocrithidia triatomae*, *Trypanosoma congolense*, and *Letomonas pyrrhocoris*^50^. The presence of parasites covering the intestinal wall of the host lowers its nutrient uptake through competition, i.e., by producing a mechanical barrier, reducing the contact surface of the microvilli, and disturbing the excretion of the host^50^. The higher number of promastigotes that effectively attach to the insect intestine may explain why females have a shorter life expectancy than males. On the other hand, the qPCR results demonstrated that there was no difference in *L. wallacei* quantification, comparing males with females. However, in qPCR we analyze both promastigotes and cysts of *L. wallacei*, whereas by microscopy, without staining the parasites, we can only see the promastigotes. Also, the classical technique for counting, using a Neubauer hemocytometer, has been employed to quantify *L. wallacei* in whole homogenates or gut contents, but the quantification of parasites in gut samples by microscopic counting is frequently hampered by the presence of a high concentration of debris in the gut, and urate crystals in the hindgut. Few reports in the literature demonstrate infection sex-bias, with notable exceptions being the fungal infections of *Metarhizium matsumae* and *M. anisopliae* in tsetse flies^5,51^.

Here, we also demonstrated an evident infection effect on insect morphology as an important aspect of *O. fasciatus* infection. Similar results have been demonstrated in the literature, and further investigation is required to investigate if morphological alterations impacts on *O. fasciatus* behavior^52,53^. In an innovative way, we delineated a straight parallel between morphology and infection of *O. fasciatus*, which may also be applied to other microorganism-host interaction studies to evaluate morphologic disturbances and determine infection statuses by morphometric parameters. The PCA and GLM data analyses applied here may reveal an opportunity for analysis of the long-term effects of infection on host morphology. Thus, these results provide evidence that *L. wallacei* severely impacts the overall morphology of its host and provides evidence that infected and uninfected insects may be distinguishable by morphometric analysis.

In parasitic infections, energy allocation may be at the center of the alterations because the host requires energy to compete with the microorganisms for nutrients and to increase resource allocation to combat the infection impacting host reproduction (Figs. S8 and S9)^43,44^^,^^54^^,^. In the present study, we also demonstrated that *L. wallacei* imposed negative effects on the reproductive fitness of *O. fasciatus*. Compared to uninfected females, a decrease in mass was observed in infected females during reproduction, although no differences in weight were observed between virgin infected and uninfected insects. Similar negative effects have been observed in response to viral infection in flies, associated with reduced rates of digestion and excretion in response to damage caused by infection^55^. *Microsporidium* infection in gypsy moth larvae results in mass loss, which is attributed to a decreased nutrient absorption^56^. Here, weight reduction in infected females could be explained by a combination of both; high number of promastigote forms in the intestine that might disturb the nutrient absorption, and the direct damage caused by infection. We also found that infected *O. fasciatus* presented decreased oviposition, egg loads, and egg eclosion. Oviposition reduction in infected insects has been reported and is associated with *Plasmodium*-mosquito interactions and trypanosomes interaction with *R. prolixus* and tsetse^57,58^. Similar observations in eclosion reduction were related to the depletion of resources for investment in egg load, which would have resulted in nutritional depletion and thus would have affected the composition of the embryo^59,60^. Similarly, we observed an increase in egg reabsorption in infected females, which suggests one reason for the observed reduction in egg load, oviposition, and viability in the infected colony, i.e., due to the reabsorption of eggs, which is a well-reported tradeoff strategy for the maintenance of somatic activities as an adaptation to infections^61–64^. Moreover, we demonstrated that infection manipulated the host *Ix* gene expression, a gene that is crucial for sexual maturation in flies^65^. In addition, when we used the RNAi protocol for the *Ix* gene, the insects developed spontaneous egg reabsorption with the same characteristics of infected insects. Thus, our data suggest that the decrease in reproductive fitness seen in infected insects is a result of egg reabsorption, which in turn is triggered by lower *Ix* gene expression. Thus, the present study argues that reproductively active females have increased infection susceptibility. This may be because females need to deal with the high energetic costs associated with the infection, which decrease the energetic stock that may be allocated for use in reproduction. Although we have evidenced innate immune activation induced by *L. wallacei*, further studies are needed to evaluate the impact of this infection on *O. fasciatus* immunity.

Regarding all the parameters that were evaluated (lifespan, reproduction, and morphology), infected insects showed decreased fitness, which posed an obvious question on whether all the modifications impact the insect population, as observed in other host-parasite models. Hence, we proposed a mathematical model to obtain short-term population projections for both colonies for comparison. The long-term behavior of the model, however, points to a well-defined reduction in population size in the infected scenario, a finding confirmed by the overall population projections. Our analysis showed exponential growth, because apart from infection, all conditions for insect development and reproduction were optimal. Readers should focus on the striking difference in population projections between infected and uninfected groups, a gap that gets bigger as time progresses. This result showed differential mortality between males and females, and was an important finding of the present study. Similar projections using a combined mortality rate for adults, i.e., models without sexual differentiation were also tested and the gap between the infected and uninfected population was albeit present. In our opinion, this constitutes an important finding, as the females bear the eggs, and it might have a differential impact on population dynamics in *O. fasciatus*.

In all scenarios tested in our mathematical models, *L. wallacei* infection can impact the population dynamics of *O. fasciatus*. Nonetheless, it is important to emphasize that our modeling approach presents two important limitations. First, it does not account for infection directly, thus it assumes that populations are either 100% infected or completely parasite-free. Although this constitutes an unrealistic assumption, as about 30% of *O.fasciatus* is infected with monoxenic trypanosomatids in nature, infection rates for this system are very difficult to measure in practice^16^. Since complete infection in a colony seems to occur within a week, our modeling results (which can span over 8 weeks) remain valid. Second, we can only obtain short-term population projections (within ~16 weeks). It would be interesting to investigate the impact of *L. wallacei* infection on the evolutionary dynamics of *Oncopeltus* populations. We hope that further studies specifically targeted at this question will be able to provide the necessary data, which (with slight model modifications), could be used to gain insights into this host-parasite interaction over more extensive temporal scales.

In conclusion, our results demonstrated the importance of a natural infection in a host’s life and suggest a new way to evaluate how infection can impact the features and fitness of the host. Also, our model could help further understanding of related insects. Finally, our study concluded that *L. wallacei* had a negative impact on its host’s physiology and that such impacts could ultimately affect *O. fasciatus* population regulation.

## Methods

### Maintenance of *O. fasciatus*

In the present study, we used two colonies of *O. fasciatus*: one was constituted of insects naturally infected with *L. wallacei* and the other was constituted of uninfected insects, which served as the control^38^. To obtain the uninfected colony, eggs collected from the infected colony were decontaminated by 2% sodium hypochlorite treatment for 5 min. After decontamination, the eggs were kept in sterile plastic containers and the newly hatched insects maintained in the same conditions described for the parental colony. In order to avoid recontamination of uninfected colony with *L. wallacei*, these insects have been kept in a different, isolated, room from the parental colony. In order to validate the absence of *L. wallacei* in the uninfected colony all the insects used in the experiments have been checked for the presence of *L. wallacei* in their guts by optical microscopy or PCR. The colonies were maintained under a 12 h light/dark cycle, at 28 °C, and at 65–75% relative humidity, as previously described^16-18,38^. All insects were supplied with sterilized peeled sunflower seeds and fresh mineral water *ad libitum*.

### Insect sorting

To obtain virgin insects in the same period of development, fifth instar nymphs were collected from parental colonies, separated into small plastic vessels. These insects were maintained in the same conditions as the parental colony. The insects were observed for the transition to the adult stage. After five days in the adult stage, the insects were used for the experiments.

### Development analysis

Insect development in both colonies was observed comparatively. Ten breeding pairs from each colony were separated and allowed to copulate for 1 week. After oviposition, 30 eggs at the same period of maturation, laid by females from both colonies, were separated into small plastic vessels. After eclosion, the insects were maintained under the same conditions as previously described. The insects were observed daily and in each developmental stage, the first and the last insect to molt were registered. With the aim of assessing if the infection influenced the mortality rate of the insects, mortalities were recorded throughout the developmental period^48^.

### Longevity of the insects

Males and females in adult stages from both colonies were maintained under the same conditions as previously described. The numbers of live and dead insects were recorded daily. The insects used in this experiment were sorted as explained above.

### Morphometric analyses

To evaluate the influence of *L. wallacei* infection to insect morphometric parameters, virgin adults and fifth instar nymphs were measured for weight, body length, total area of the body, membranous wing and hemielytra length, membranous wing and hemielytra area, rostrum and median leg length, abdomen width and antenna length. The insects were photographed on graph paper and measured using the software Analyzing Digital Images (program provided by Museum of Science, Boston, MA, USA).

### Reproductive analysis

Oviposition and egg load were observed in individually separated copulated, infected and non-infected females. The oviposition was observed for two weeks. Oviposited eggs from both colonies were separated in glass vials and counted daily for eclosion. To observe egg reabsorption and egg load, females were collected after the copulation period and their ovaries were dissected in order to perform morphologic evaluations of the ovarian follicles and for egg counting. Eggs were considered to be in the reabsorption process when possessing an opaque ooplasm and a loss of shape of ellipsoid prolate, which is otherwise a characteristic of normal eggs^61^. All insects were weighed using a precision scale.

### *Intersex* gene expression

Ovaries of insects from both colonies (infected and uninfected n = 24) were dissected in PBS, pH 7.4. RNA was extracted with Trizol (Invitrogen), following the manufacturer’s protocol. RNA subsamples (1 μg) were treated with 1 U DNase (Fermentas) in DNA buffer in a total volume of 10 μl and incubated at 37 °C for 30 min. The samples were then incubated at 65 °C for 10 min with 1 μl EDTA 4.5 mM for DNase inactivation. Then, a high-capacity cDNA reverse transcription kit (Applied Biosystems) was used for cDNA synthesis, following the manufacturer’s protocol. The qPCR for intersex expression was performed for cDNA qualitative analysis in a final volume 15 μl of 7,5 μl power SYBR green PCR Master Mix (Applied Biosystems), 5 μl cDNA (1:10 diluted), and 350 nM primers: forward: 5′-GAGTAGCCCCGACGAGAAGT-3′ and reverse 5′-ATGCCATGCATTTCCTTAGC-3′. The qPCR reactions were performed using a StepOnePlus Real-Time PCR System (Applied Biosystems), with default settings: 10 min at 95 °C, 40 cycles of 15 s at 95 °C, and 1 min at 60 °C, and finally by a denaturation curve. The ΔΔCt method was comparatively used to analyze the alterations in gene expression. Specific primers for eIF3: forward 5′-CTTCAGCTTCTTTGGGTTGG-3′ and reverse 5′-GAAATGTGGGAAGACCGAGA-3′ were used to normalize the expression levels of the *intersex* gene.

### RNAi protocol

*Intersex* gene silencing protocol was performed by RNAi silencing by dsRNA administration by feeding. For specific intersex dsRNA (dsix) synthesis, gene segments were generated by PCR using primers: forward: 5′-TAATACGACTCACTATAGGGCCACCTCAGGAGAAACTGGA-3′ and reverse: 5′-TAATACGACTCACTATAGGGATGCCATGCATTTCCTTAGC-3′. Those templates were used for dsRNA and in the Kit T7 MEGAscript (Thermo Fischer), following the manufacturer’s protocol. The dsRNA was then quantified by Nanodrop 1000 v.3.7 (Thermo Fisher Scientific) and dsRNA dissolved in sterile water for insect feeding. For the control group, dsmal was offered to the insects via the water. The dsmal was synthesized as reported for dsix using a template for plasmid Litmus 28i-mal and primers: forward and reverse: 5′-TAATACGACTC ACTATAGGG-3′. Third stadium nymphs (n = 30) were separated and monitored until they reached the forth stadium. The insects were then fasted for two days, after which they were offered the dsix and dsmal in the water for two more days. After reaching the adult stage, the inhibition of the intersex gene expression was evaluated by qPCR as described in the previous section.

### Conventional PCR and qPCR for parasite detection

Primers specific for *L. wallacei* and *O. fasciatus* 16 S rRNA gene were designed as previously described^38^. The sequences of the primers designed are as follows: F-Lw 5′-CTTTTGGTCGGTGGAGTGAT-3′ and R-Lw 5′-GGACG-TAATCGGCACAGTTT-3′; F-Of 5′-CAAAATTTGGTTGGGGTGAC-3′ and R-Of 5′-ATC-GAGGGTCGCAAACTCTT-3′. Total RNA was extracted using Trizol (Invitrogen) following the manufacturer’s protocol. We have treated the RNA with RNase-free DNase I (Fermentas International Inc., Burlington, Canada), and cDNA was synthesized using the High Capacity cDNA reverse transcription kit following manufacturer’s protocol (Applied Biosystems, Foster City, CA). cDNA from whole intestine was PCR-amplified using the PCR master mix (Fermentas International Inc.). The amplification reactions were performed as previously reported by our group^38^ in a final volume of 10 μl. Each reaction was performed with 50 ng of DNA sample, 5 μl of PCR Master Mix (Fermentas International Inc., Burlington, Canada) and 350 mM of primers specific for *L. wallacei* or *O. fasciatus*. The PCR was performed as follows: initial denaturation of DNA for 5 min at 94 °C; 40 amplification cycles each consisting of 30 sec at 94 °C, 45 sec at 53 °C for both parasite and insect DNA amplification and 30 sec at 72 °C; and a final step of 5 min at 72 °C for extension of incomplete products. Following PCR, the amplification products were analyzed by electrophoresis in 2% (wt/v) agarose gels that were submitted to ethidium bromide staining and analyzed under ultraviolet light excitation in comparison to GeneRuler TM 100 bp Plus DNA ladder fragments (Fermentas International Inc.). qPCR was performed on a StepOnePlus real-time PCR system (Applied Biosystems) using the Power SYBR Green PCR master mix (Applied Biosystems). We used comparative Ct method to compare gene expression levels and the *O. fasciatus* 16 S rRNA gene was used as an endogenous control.

### Number of promastigotes in the intestine

Adult insects from the infected colony were collected and dissected in 200 µl phosphate-buffered saline (PBS, pH 7.4) and the promastigote forms counted in a Neubauer chamber under a Zeiss Axioplan 2 light microscope (Oberkochen, Germany). Each intestinal compartment was macerated in PBS and the number of live promastigotes counted for each insect separately, according to the gut region.

### Scanning electron microscopy

Insect guts were dissected in PBS at 4 °C before fixation. Fixation involved immersion in a solution that contained 2.5% glutaraldehyde, 4.0% formaldehyde, 3.7% of sucrose, and 5 mM CaCl_2_ in 0.1 M cacodylate buffer (pH 7.2) for 2 h at 26 °C. After three washes in 0.1 M cacodylate buffer (pH 7.2), samples were dehydrated using an ethanol series (50, 70, 90, and 100%) and dried using the critic point method in a Balzers CDP-20 apparatus (Balzers Union, Fürstentun Liechstenstein). The micrographs were made using a scanning electron microscope (Jeol JSM-5310).

### Statistical analysis and mathematical modeling

We employed a Weibull regression model to estimate survival curves with confidence bands for the four experimental groups (infected males, uninfected males, infected females, and uninfected females). Morphometric data were first analyzed using a principal component analysis (PCA) and then the first principal component (PC) (which retained most of sampling variance) was used as the overall size indicator. This size variable and the insect sex were used as predictors of infection in a binary generalized linear model (GLM) with a logit link function. As many of the daily laid egg counts were zero, a zero-inflated Poisson GLM was employed to model oviposition through time for infected and uninfected females. Posterior distributions for the proportions of egg eclosion and re-absorption were obtained using a binomial likelihood with a conjugate Beta (1, 1) prior.

To integrate all the data collected in this study in a coherent manner, we used data on reproductive fitness and development to parameterize a system of ordinary differential equations that model *Oncopeltus* population growth. Our models were age-structured and we explored models with and without sexual differentiation of adults. Models with sexual differentiation were employed to capture differences in mortality between males and females. The governing equations for the model with sexual differentiation were:

$$E^{\prime} =oF-eE$$

$${{N}_{1}}^{^{\prime} }=eE-{d}_{1,2}{N}_{1}-{m}_{1}{N}_{1}$$

$${{N}_{j}}^{^{\prime} }={d}_{j-1,i}{N}_{j-1}-{d}_{j,j+1}{N}_{j}-{m}_{j}{N}_{j},j=2,\ldots ,5$$

$$M^{\prime} =(1-{p}_{F}){d}_{5,A}{N}_{5}-{m}_{M}M$$

$$F^{\prime} ={p}_{F}{d}_{5,A}{N}_{5}-{m}_{F}F$$

A description of the parameters can be found in Data S1 and Table S3. We used the data collected in this study to construct two sets of parameters, one for an infected and another for an uninfected population scenario. Using this model and a different set of parameters for each scenario we obtained short-term population projections under both scenarios and then compared the resulting trajectories. Further theoretical background on the statistical and mathematical analyses is provided in S1 Data. The R code to perform all the described statistical and mathematical analyses is publicly available at https://github.com/maxbiostat/CODE/tree/master/OncoLeptoModeling.

## Supplementary information

Dataset 1

Related Manuscript File
