# Supplementary material for: Natural infection by the protozoan Leptomonas wallacei impacts the morphology, physiology, reproduction, and lifespan of the insect Oncopeltus fasciatus
Source: Sci Rep. 2019 Nov 25;9:17468. doi: 10.1038/s41598-019-53678-1 (PMC6877526; doi:10.1038/s41598-019-53678-1)
Supplement: Supplementary file 1 — Dataset 1 [file 41598_2019_53678_MOESM1_ESM.pdf]

S1 DATA

Natural infection by the protozoan *Leptomonas wallacei* impacts the morphology, physiology, reproduction, and lifespan of the insect *Oncopeltus fasciatus*

Luiz Ricardo C. Vasconcellos, Luiz Max F. Carvalho, Fernanda A. M. Silveira, Inês C. Gonçalves, Felipe S. Coelho, Octávio A. C. Talyuli, Thiago L. Alves e Silva, Leonardo S. Bastos, Marcos H. F. Sorgine, Leonan A. Reis, Felipe A. Dias, Claudio J. Struchiner, Felipe Gazos-Lopes, Angela H. Lopes

1 Reproductive fitness

Reproductive fitness was evaluated by observing oviposition, egg eclosion and egg reabsorption. Oviposition was measured by simply observing the number of eggs laid, on a daily basis, for two weeks for both groups. We then modeled oviposition using a zero-inflated Poisson generalized linear model (GLM) [1, 2]. First, for each female F_i, consider an indicator function $\sigma(F_i)$ that is 1 if the female oviposited and 0 otherwise. Next, assume the number of eggs laid (n_i) follows a Poisson distribution. Thus,

$$\sigma(F_i) \sim Degenerate(\theta_i), \tag{1}$$

$$n_i \sim Pois(\lambda_i). \tag{2}$$

We assumed that the observed zeroes come from two distinct data-generating processes: a proportion θ_i comes from a zero inflation process and a proportion $1 - \theta_i$ comes from a Poisson distribution with parameter λ_i. The number of eggs per female, E_i, has probability mass function:

$$P(E_i = k) = \begin{cases} \theta_i + (1-\theta_i)e^{-\lambda_i} & E_i = 0, \\ (1-\theta_i)\frac{e^{-\lambda_i}\lambda_i^k}{k!} & E_i > 0. \end{cases} \tag{3}$$

Finally, suppose some covariates $z_{ij} = \mathbf{z}, j = 1, 2, .., k$ were measured. We then have[1]

$$\log(\lambda_i) = \mathbf{z}^\intercal \boldsymbol{\beta}^\lambda, \tag{4}$$

$$\log\left(\frac{\theta_i}{1-\theta_i}\right) = \mathbf{z}^\intercal \boldsymbol{\beta}^\theta. \tag{5}$$

The GLM formulation allows us to model the dependency of E_i on both time and infection status. Models were fitted using the **pscl** package [3] of the R statistical computing

[1]Notice that, although here we make both λ_i and θ_i depend on the $\mathbf{z}$ covariate matrix, this does not need to be so. One could as well model the two parameters on different sets of covariates.

environment[4]. Predictions for oviposition in both groups are presented in Fig. 4B in main text.

Eclosion was determined by counting the number of eggs ecloded, which in turn allows for estimating a proportion of eclosion, ρ^e, for each group (infected and uninfected). To estimate ρ^e, we formulate our model as follows: for each infection status k, let Y_{ki} be an indicator variable for the i-th egg such that

$$Y_{ki} = \begin{cases} 1 & \text{if the egg ecloded,} \\ 0 & \text{if the egg did not eclode.} \end{cases} \tag{6}$$

Next, define $x_k = \sum_i^{n_k} Y_{ki}$ to be the counts of eggs ecloded over n_k observed. Bayesian inference about ρ_k^e is done by assuming

$$Y_{ki} \sim Bern(\rho_k^e), \tag{7}$$

$$x_k \sim Bin(n_k, \rho_k^e). \tag{8}$$

Thus, the likelihood function is

$$p(x_k|\rho_k^e) = \binom{n_k}{x_k} (\rho_k^e)^{x_k} (1 - \rho_k^e)^{n_k - x_k} \tag{9}$$

To complete inference about ρ_k^e, we need to specify a prior distribution for it:

$$\rho_k^e \sim Beta(a, b). \tag{10}$$

Here, we set $a = b = 1$ to obtain an uniform prior. By Bayes theorem, we know that

$$p(\rho_k^e|x_k) \propto p(x_k|\rho_k^e)p(\rho_k^e), \tag{11}$$

and then arrive at the posterior

$$p(\rho_k^e|x_k) \propto (\rho_k^e)^{x_k + a - 1}(1 - \rho_k^e)^{b + n_k - x_k - 1}, \tag{12}$$

which is a Beta distribution with parameters $x_k + a$ and $b + n_k - x_k$. Results of the eclosion experiment are presented in S1 Table.

S1 Table: Egg eclosion per infection status.

	Infected	Uninfected
Ecloded	91	95
Non-ecloded	29	14

From these results we could estimate eclosion proportions (and 95 % credible invervals) for both groups, resulting in $\rho_I^e = 0.76$ $(0.67, 0.83)$ and $\rho_U^e = 0.87$ $(0.83, 0.95)$.

Ovary atresy was observed by dissecting females and counting the number of vitelogenic eggs (see S2 Table). Proportions of reabsorbed eggs (ρ^r) were computed in the same fashion outlined above.

Using these data, we estimated $\rho_I^r = 0.06$ $(0.04, 0.08)$ and $\rho_U^r = 0.01$ $(0, 0.01)$.

S2 Table: Ovary atresy (egg reabsorbtion) per infection status.

	Infected	Uninfected
Reabsorbed	38	3
Vitelogenic	618	588

2 Survival

To assess the impact of infection on insect survival, we observed 75 adult insects (45 infected, 30 uninfected), separated by sex (30 males, 45 females) until death. For each group (infected males (MI); uninfected males (MU); infected females (FI); uninfected females (FU)), we are interested in the time to death. Let the time to death be a continuous[2] random variable, T, with distribution function $F(t) = P(T \leq t)$ and probability density function $\frac{dF(t)}{dt} = f(t)$. We can therefore define a simple survival function $S(t) = P(T > t) = 1 - F(t)$. One may be interested in knowing the probability of death at a particular time t conditional on survival to that time. This is called the *harzard function*, $h(t)$ and can obtained by taking the limit

$$h(t) = \lim_{\Delta t \to 0} \frac{P(t \leq T < t + \Delta t)}{\Delta t}, \tag{13}$$

$$= \frac{f(t)}{S(t)}. \tag{14}$$

In what follows, we assume T is Weibull distributed with parameters θ and γ (shape and scale, respectively). This gives the hazard function

$$h(t) = \gamma \theta t^{\gamma - 1}. \tag{15}$$

Also, considering that for each individual in the study we also observe some covariates $\mathbf{z}$, which here are infection status and sex. Then we can model θ as linear combination of $\mathbf{z}$, in a GLM fashion. Since we want T to have support only on $[0, \infty)$, we make $log\theta = \beta_0 + \mathbf{z}^\intercal \boldsymbol{\beta}$, where $\boldsymbol{\beta}$ is a vector of regression coefficients. Thus

$$h(t|\mathbf{z}) = \gamma \exp(\beta_0 + \mathbf{z}^\intercal \boldsymbol{\beta}) t^{\gamma - 1}. \tag{16}$$

For a desired level of confidence α, $100\alpha\%$ confidence bands for $S(t|\mathbf{z})$ using the relation:

$$S(t|z_i) = \exp\left(- \left(\frac{t}{e^{\theta_i}} \right)^{\frac{1}{\gamma}} \right). \tag{17}$$

After obtaining $100\alpha\%$ confidence intervals for each θ_i using standard methods, one can plug in these values, along with the estimate for γ, into equation 17. For details see [5] and [6]. We used the R package **survival** [7] to fit this model to data and prediction curves for each group are presented in Fig. 1A (main text).

[2] This assumption is made solely for simplicity.

3 Morphometric analysis

In order to understand the impact of *Leptomonas* infection on the insects' phenotype, several morphometric attributes were analyzed, for infected and uninfected groups. Comparisons were also made by sex. In total, 11 attributes were measured for adult insects and 7 for nymphs, including wings (forewing and hindwing) area, total length, hemi-elytra length, leg length, rostrum length and insect weight. Details can be found in the Methods section of the main text.

We then applied Principal Components Analysis (PCA) to both data sets in order to reduce dimensionality and avoid multicolinearity. Consider observing p – possibly correlated – continuous attributes of N individuals. PCA allows us to transform the data $N \times p$ matrix $\mathbf{D_p}$ into a set $\boldsymbol{x}$ of p new orthogonal vectors, the so-called the principal components (PCs). This is done by obtaining linear combinations of the columns of $\mathbf{D_p}$ so as to maximize variance of these new vectors. The main advantage of this approach is that the new vectors are uncorrelated, and we usually need to retain far less than p PCs to achieve a satisfactory description of the variation in the data.
For both data sets – adults and nymphs – PCA resulted in a first principal component (PC) that explains about 70% of the total variance and is positively correlated with all the original variables. We took this PC to describe insect size in general. To assess the association of the obtained principal component with infection status, we again took a generalized linear model (GLM) approach. The model was of the form:

$$Y_i \sim \text{Bernoulli}(p_i), \tag{18}$$

$$\log\left(\frac{p_i}{1-p_i}\right) = \mathbf{x_i^\intercal}\boldsymbol{\beta} + \epsilon_i, \tag{19}$$

where $\boldsymbol{\beta}$ is the vector of regression coefficients and $\mathbf{x}$ is the matrix of covariates, in this case insect sex and the size-related PC. The errors $\epsilon \sim \mathcal{N}(0, \sigma^2)$ with σ^2 known. For these analyses we used the package **arm** in R to fit a model with interaction between insect sex and size having infection status as a response variable.

4 Mathematical modeling

Here, some models were developed to help us understand the growth dynamics of infected and uninfected populations of *Oncopeltus fasciatus*. We built differential equations-based models to obtain projections for insect populations in the presence and absence of infection. We capitalize on the data collected and analyzed in the previous sections to parameterize our models.

In this section we present the system of ordinary differential equations proposed to model *Oncopeltus fasciatus* population growth in more detail. Although the infection dynamics is also of interest, in the laboratory environment the infection spreads so fast we cannot accurately measure transmission rates. Thus, instead of coupling infection and demography, we chose to develop a purely demographic model and used different parameter values (based on the available data) for the infected and uninfected groups to create different scenarios. In the compartmental model developed here each developmental stage of the insect (eggs,

nymphs, adults) was modeled as a state variable. Let E be the state variable for the eggs laid and $N_i, i = 1, 2, ..., 5$ represent the nymph stages and A represent the adult stages. Denote $\frac{dX}{dt} := X'$. The governing equations are:

$$E' = o\left(\frac{A}{2}\right) - p_e e E \tag{20}$$

$$N_1' = p_e e E - d_{12} N_1 - m_1 N_1 \tag{21}$$

$$N_j' = d_{j-1,i} N_{j-1} - d_{j,j+1} N_j - m_j N_j, \forall j = 2, ..., 5 \tag{22}$$

$$A' = d_{5,A} N_5 - m_A A \tag{23}$$

A schematic representation of this system is given in S9A Fig. One possible alternative structure for this demographic model is to allow for differential sexual maturation and mortality, in turn modeling males and females as separate compartments. We can then study the possible effects of differential sexual response to infection. Thus the alternative equations for the system are:

$$E' = oF - p_e e E \tag{24}$$

$$N_1' = p_e e E - d_{1,2} N_1 - m_1 N_1 \tag{25}$$

$$N_j' = d_{j-1,i} N_{j-1} - d_{j,j+1} N_j - m_j N_j, \forall j = 2, ..., 5 \tag{26}$$

$$M' = (1 - p_F) d_{5,A} N_5 - m_M M \tag{27}$$

$$F' = p_F d_{5,A} N_5 - m_F F \tag{28}$$

where p_F is the probability of a nymph turning into a female[3], and we furthermore allow differential sexual mortality rates, m_M and m_F. S9B Fig. shows the diagram for this model with sexual differentiation (SD). Please see S3 Table for a complete description of model parameters. Clearly, when $p_F = 0.50$ and $m_M = m_F = m_A$, the model in equations (24–28) becomes the system presented in (20–23).

Several questions regarding population biology can be studied using the models presented here. We can, for instance, compare the population growth under the reference (no infection) and an infection scenario.

[3]Which should correspond approximately to the proportion of females in the population.

S3 Table: Parameters of the differential equations models presented at equations (20–23) and (24–28). Parameter values were obtained from the data collected and analyzed in the above sections, otherwise stated. * – parameter value obtained from the literature.

Parameter	Description	Uninfected	Infected
o	oviposition rate (day^{-1})	21	12
p_e	eclosion probability	0.87	0.76
e	eclosion rate (day^{-1})	0.17	0.14
$d_{1,2}$	development rate from N_1 to N_2 (day^{-1})	0.62	0.38
$d_{2,3}$	development rate from N_2 to N_3 (day^{-1})	0.2	0.36
$d_{3,4}$	development rate from N_3 to N_4 (day^{-1})	0.45	0.21
$d_{4,5}$	development rate from N_4 to N_5 (day^{-1})	0.2	0.38
$d_{5,A}$	development rate from N_5 to adults (A) (day^{-1})	0.22	0.1
m_1	mortality rate of N_1 (day^{-1})	0.33	0.33
m_2	mortality rate of N_2 (day^{-1})	0.33	0.33
m_3	mortality rate of N_3 (day^{-1})	0.33	0.33
m_1	mortality rate of N_4 (day^{-1})	0.33	0.33
m_1	mortality rate of N_5 (day^{-1})	0.5	0.5
m_A	mortality rate of adults (day^{-1})	0.01	0.03
m_M	mortality rate of males (day^{-1})	0.01	0.02
m_F	mortality rate of females (day^{-1})	0.02	0.05
p_F	probability of becoming a female adult*	0.55	0.55

We can compare a disease-free (uninfected) and an infected scenario by solving the system of differential equations with different sets of parameter values (see S3 Table) and then plotting the ratio infected/uninfected for each compartment (stage of development). We show the results for the models without and with sexual differentiation in S8 Fig. and Fig. 6 (main text), respectively.

References

[1] Lambert D. Zero-inflated Poisson regression, with an application to defects in manufacturing. Technometrics. 1992;34(1):1–14.

[2] MacCullagh P, Nelder JA. Generalized linear models. vol. 37. CRC press; 1989.

[3] Zeileis A, Kleiber C, Jackman S. Regression Models for Count Data in R. Journal of Statistical Software. 2008;27(8). Available from: http://www.jstatsoft.org/v27/i08/.

[4] R Core Team. R: A Language and Environment for Statistical Computing. Vienna, Austria; 2013. Available from: http://www.R-project.org/.

[5] Borgan O, Gjessing HK, Gjessing S. Survival and event history analysis: a process point of view. Springer; 2008.

[6] Kleinbaum DG, Klein M. Survival analysis. Third edition ed. Springer; 2012.

[7] Therneau TM. A Package for Survival Analysis in S; 2013. R package version 2.37-4. Available from: http://CRAN.R-project.org/package=survival.
