# Supplementary material for: Natural infection by the protozoan Leptomonas wallacei impacts the morphology, physiology, reproduction, and lifespan of the insect Oncopeltus fasciatus
Source: Sci Rep. 2019 Nov 25;9:17468. doi: 10.1038/s41598-019-53678-1 (PMC6877526; doi:10.1038/s41598-019-53678-1)
Supplement: Supplementary file 2 — Related Manuscript File [file 41598_2019_53678_MOESM2_ESM.pdf]

Natural infection by the protozoan *Leptomonas wallacei* impacts the morphology, physiology, reproduction, and lifespan of the insect *Oncopeltus fasciatus*

Luiz Ricardo C. Vasconcellos, Luiz Max F. Carvalho, Fernanda A. M. Silveira, Inês C. Gonçalves, Felipe S. Coelho, Octávio A. C. Talyuli, Thiago L. Alves e Silva, Leonardo S. Bastos, Marcos H. F. Sorgine, Leonan A. Reis, Felipe A. Dias, Claudio J. Struchiner, Felipe Gazos-Lopes, Angela H. Lopes

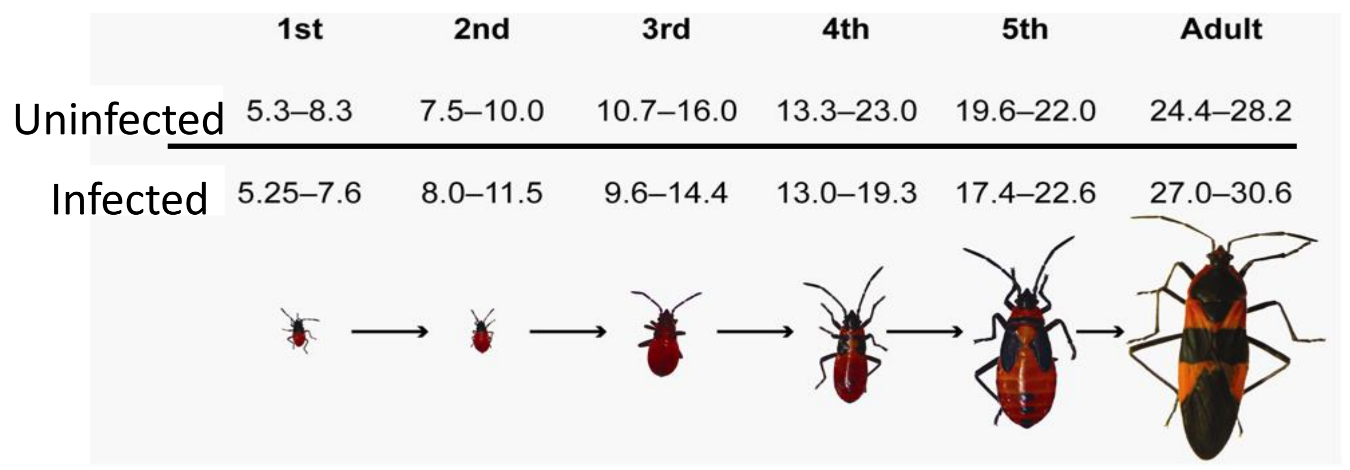

Supplemental Figure 1. Minimum and maximum time in days for infected and uninfected *Oncopeltus fasciatus* reach their developmental stages. Insect development was evaluated from egg eclosion until reaching the adult stage. Time in days required to achieve each life stages was observed and computed for insects from both colonies. *Oncopeltus fasciatus* has five nymphal forms, from the newly hatched nymph till the fifth instar, as represented in the images. Above each representative figure of developmental stages is shown the minimum (left) and maximum (right) period of time spent in each stage.

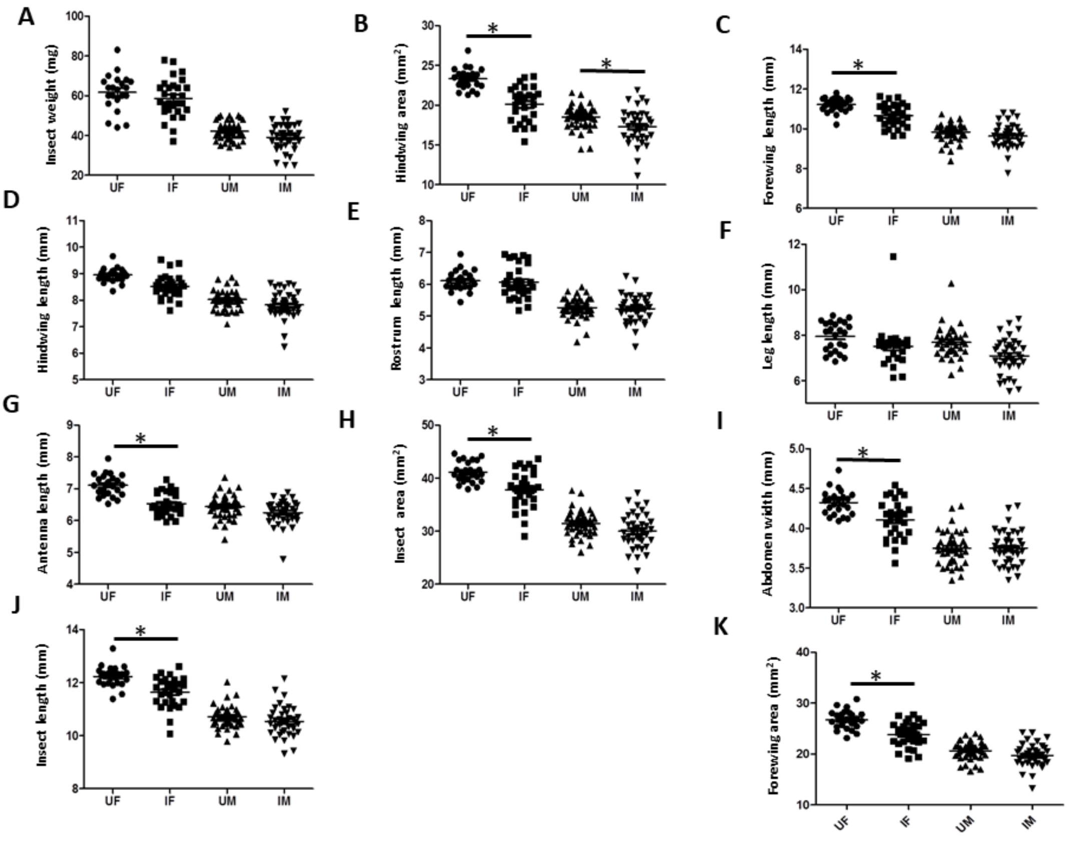

Supplemental Figure 2. *Leptomonas wallacei* infection induces morphological alterations in *Oncopeltus fasciatus*. Adult insects were collected from infected and uninfected colonies and their morphologies were analyzed. The insects were compared by weight (A) weight, (B) length, (C) forewing length, (D) hindwing length, (E) rostrum length, (F) leg length, (G) Antenna length, (H) Insect area, (I) Abdomen width, (J) insect length and (K) forewing area. UF = uninfected females, IF = infected females, UM = uninfected males and IM = infected males.

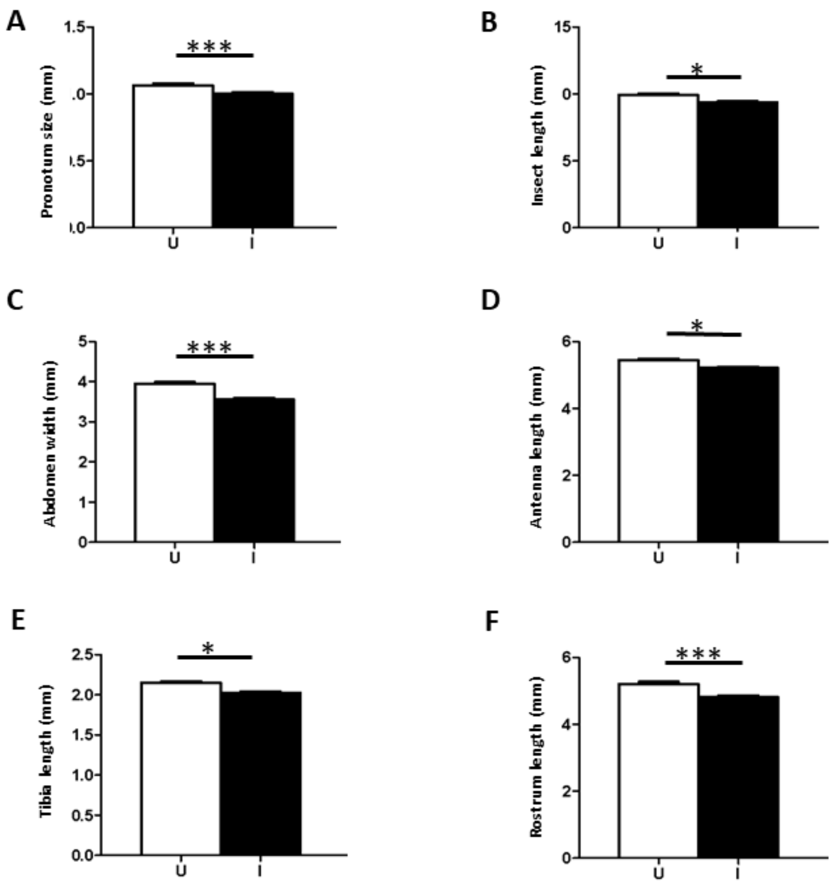

Supplemental Figure 3. *Leptomonas wallacei* infection induces alterations in nymph morphology. Nymphs in fifth instar from infected and uninfected colonies were collected and their morphologies were analyzed. The pronotum size (A), insect length (B), abdomen width (C), antenna length (D), tibia II length (E), and rostrum length (F) were compared between insects. I = infected nymphs, U = uninfected nymphs. * p > 0,05 and *** p> 0,001 in student's t-test

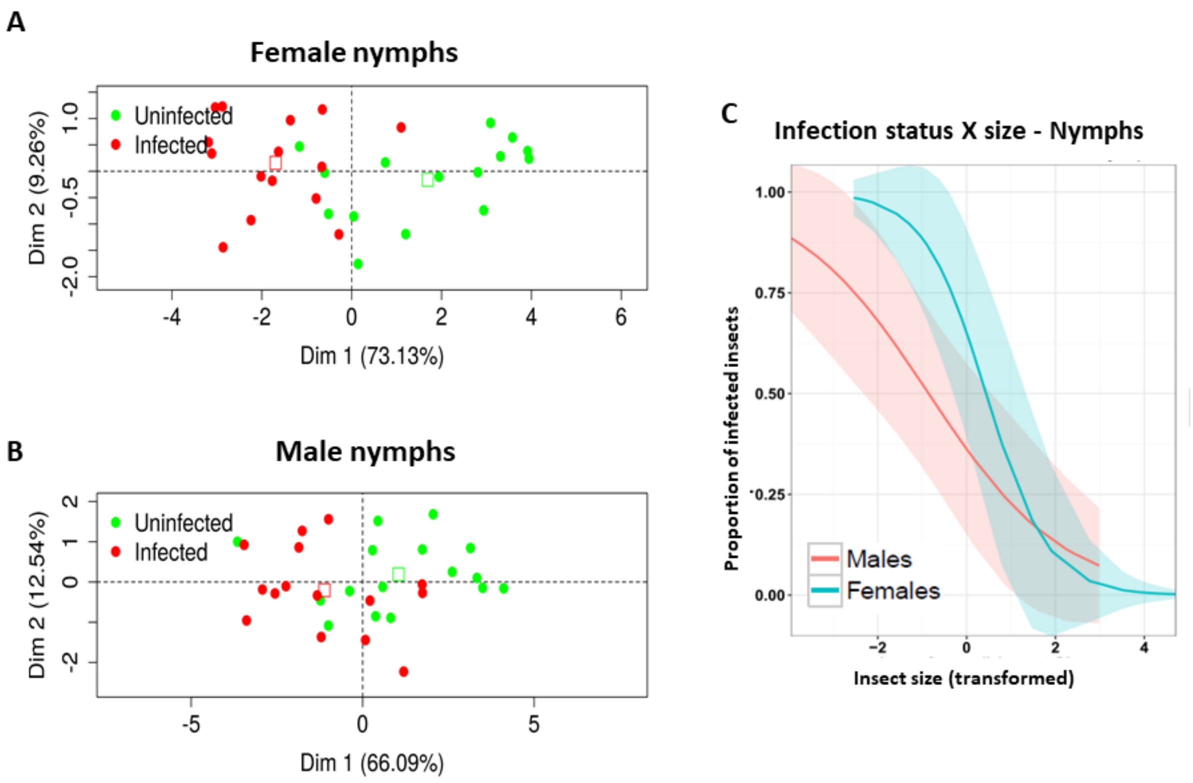

Supplemental Figure 4. Principal components analysis (PCA) plots for the morphometric data in nymphs. All morphometric variables were transformed using the PCA, and plots showing the first and second principal components and each data point graphically represents a sample of (A) females and (B) females (infected-red dots and uninfected-green dots). (C) Generalized linear model (GLM) predictions of the probability of being infected in nymphs. Results of GLM predictions of the probability of being infected in response to the size principal component derived using the PCAs for males and females and the nymph dataset.

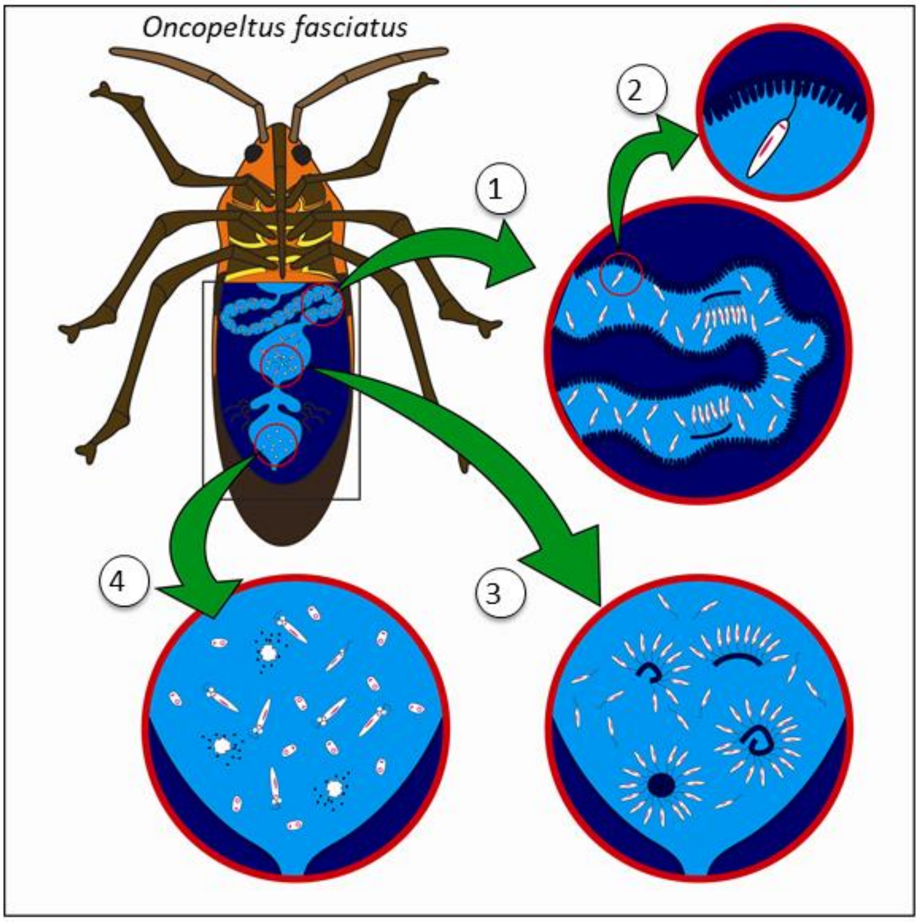

Supplemental Figure 5. ***Leptomonas wallacei*** **infection cycle in** ***Oncopeltus fasciatus*****.** The insect ingests *L. wallacei* and the parasite passes through alimentary tract of the insect; through the foregut until it reaches the midgut *L. wallacei* where it binds to the perimicrovillar membranes and replicates by cell division (1 and 2). Linked to the perimicrovillar membranes via their flagella, the *L. wallacei* are carried up to hindgut, where encystment occurs through unequal division (3). The cystic forms may be released in the insect excreta, and other insects may ingest these forms through feeding, which gives rise to a new *L. wallacei* cycle (4). This image was drawn by Rodrigo Heleno do Nascimento da Silva.

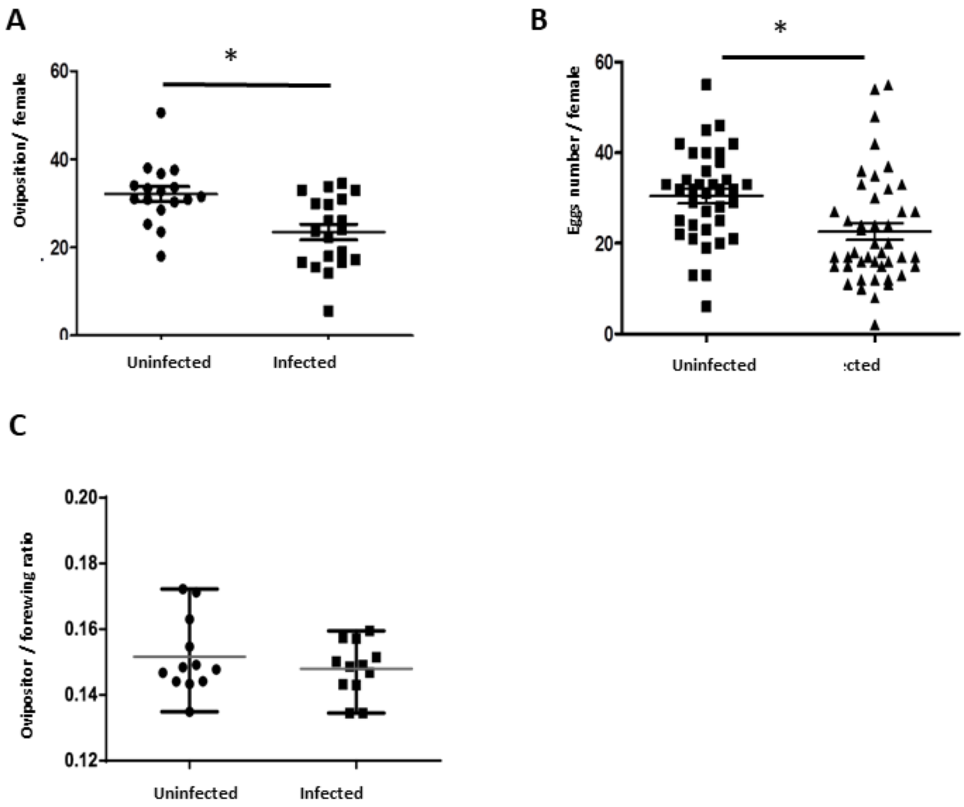

Supplemental Figure 6. *Leptomonas wallacei* infection induces reproductive fitness reduction on *Oncopeltus fasciatus*. (A) Oviposition was evaluated in females from infected and uninfected colony (B) Reproductively active females from both colonies were dissected and the egg load (number of eggs produced) was counted. (C) Ovipositor size normalized with forewing length in infected and uninfected females. U = uninfected and I = infected. F = female and M = male. * p > 0,05 in the student's t-test.

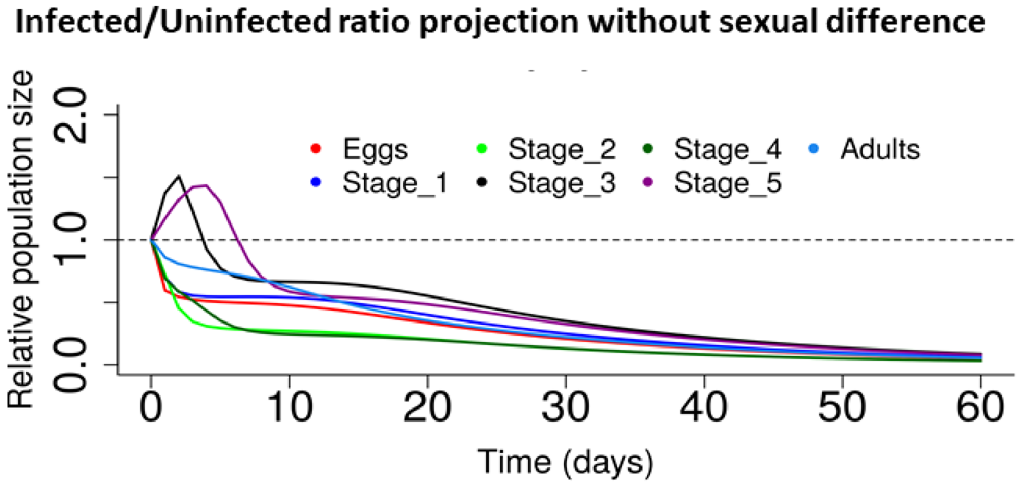

Supplemental Figure 7. Diagrammatic representations of a demographic model. We show the diagram of the simplest demographic model, without sexual dimorphism.
